# Electromagnetic Enhancement of Graphene Raman Spectroscopy by Ordered and Size-Tunable Au Nanostructures

**DOI:** 10.1186/s11671-015-1098-6

**Published:** 2015-10-06

**Authors:** Shuguang Zhang, Xingwang Zhang, Xin Liu

**Affiliations:** State Key Laboratory of Luminescent Materials and Devices, South China University of Technology, Guangzhou, 510641 China; Key Lab of Semiconductor Materials Science, Institute of Semiconductors, CAS, Beijing, 100083 China

**Keywords:** Surface plasmons, Graphene, Ordered Au nanostructures, Raman scattering

## Abstract

The size-controllable and ordered Au nanostructures were achieved by applying the self-assembled monolayer of polystyrene microspheres. Few-layer graphene was transferred directly on top of Au nanostructures, and the coupling between graphene and the localized surface plasmons (LSPs) of Au was investigated. We found that the LSP resonance spectra of ordered Au exhibited a redshift of ~20 nm and broadening simultaneously by the presence of graphene. Meanwhile, the surface-enhanced Raman spectroscopy (SERS) of graphene was distinctly observed; both the graphene G and 2D peaks increased induced by local electric fields of plasmonic Au nanostructures, and the enhancement factor of graphene increased with the particle size, which can be ascribed to the plasmonic coupling between the ordered Au LSPs and graphene.

## Background

Graphene is the first two-dimensional carbon atomic crystal which is constructed by several layers of honeycomb-arrayed carbon atoms. This promising material is highly attractive for the fabrication of high-frequency nanoelectronic and optoelectronic devices due to its exceptional optical and electrical properties, such as extreme mechanical strength, ultrahigh electrical carrier mobility, and very high light transmittance [1–3]. Unfortunately, the graphene of only one-atomic-layer thickness exhibits lower light absorption (only ~2.3 % for a single layer) originating from the weak light-graphene interaction, which is unfavorable for high-performance graphene-based optoelectronic devices. Several approaches have been proposed to enhance the absorption of graphene, including using one-dimensional photonic crystal and localized surface plasmons (LSPs) [4, 5]. LSPs in conventional systems are the collective oscillations of conduction electrons in the metal nanoparticles when illuminated and excited by light with appropriate wavelength, and the resonance excitation of the LSPs induces a large enhancement and confinement of the local electric field in the vicinity of the metal nanostructures. Generation of LSPs stimulates a wide range of applications such as ultratrace biochemical sensing, enhanced absorption in photovoltaic cells, surface plasmon-enhanced fluorescence, and Raman scattering. From a spectroscopic point of view, surface-enhanced Raman spectroscopy (SERS) has become a promising spectacular application of plasmonics especially for the graphene-LSP hybrid system. On the other hand, the two-dimensional nature of graphene and its well-known Raman spectrum make it a favorable test bed for investigating the mechanisms of SERS, and various nanoparticle geometries have proven to deliver a considerable Raman enhancement in the case of graphene [6–11]. A Raman enhancement of 103 times had been detected for graphene from the dimer cavity between two closely packed Au nanodisks. However, fabrication and space control of the Au nanodisks are very complex and costly [6]. Sun et al. deposited Ag on the surface of a graphene film, and distinct Raman enhancement had been achieved. However, the quality of graphene significantly deteriorated after deposition of metal nanoparticles which limits the further application of graphene [8].

Except for enhancing intensity of Raman scattering of graphene by LSPs, graphene had also been adopted to tune the surface plasmons resonance wavelength of metal nanostructures. For instance, the plasmonic behavior of Au nanoparticles can be tuned by varying the thickness of the Al_2_O_3_ spacer layer inserted between the graphene and nanoparticles [12]. Nevertheless, the Au nanoparticles are randomly distributed on the surface of the Al_2_O_3_ layer which is unfavorable for the precise controllability and investigation of the inter-coupling of the graphene-metal hybrid system. Obviously, combination of enhanced near-fields of ordered plasmonic nanostructures with unusual optoelectronic properties of graphene will provide a more promising application for novel graphene-based optoelectronic devices, and thus, research on the plasmonic coupling between graphene and plasmonic ordered nanoparticles is highly desirable.

In this paper, ordered and size-controlled Au nanostructures were fabricated using the inverted self-assembled monolayer template of polystyrene microspheres. A chemical vapor deposition (CVD) graphene was transferred directly on top of Au nanostructures, and the interaction between graphene and LSPs of Au has been systematically investigated. We found that the SERS of graphene was apparently observed and Raman intensities of both the graphene G and 2D peaks increased with the size of Au which was induced by the local electric field of plasmonic Au nanostructures. On the other hand, the absorption spectra of Au nanostructures exhibited a redshift of ~20 nm and a slight broadening by the presence of graphene, which was due to the inter-coupling between Au LSPs and graphene.

## Methods

### Sample Preparation

**Fig. 1:**
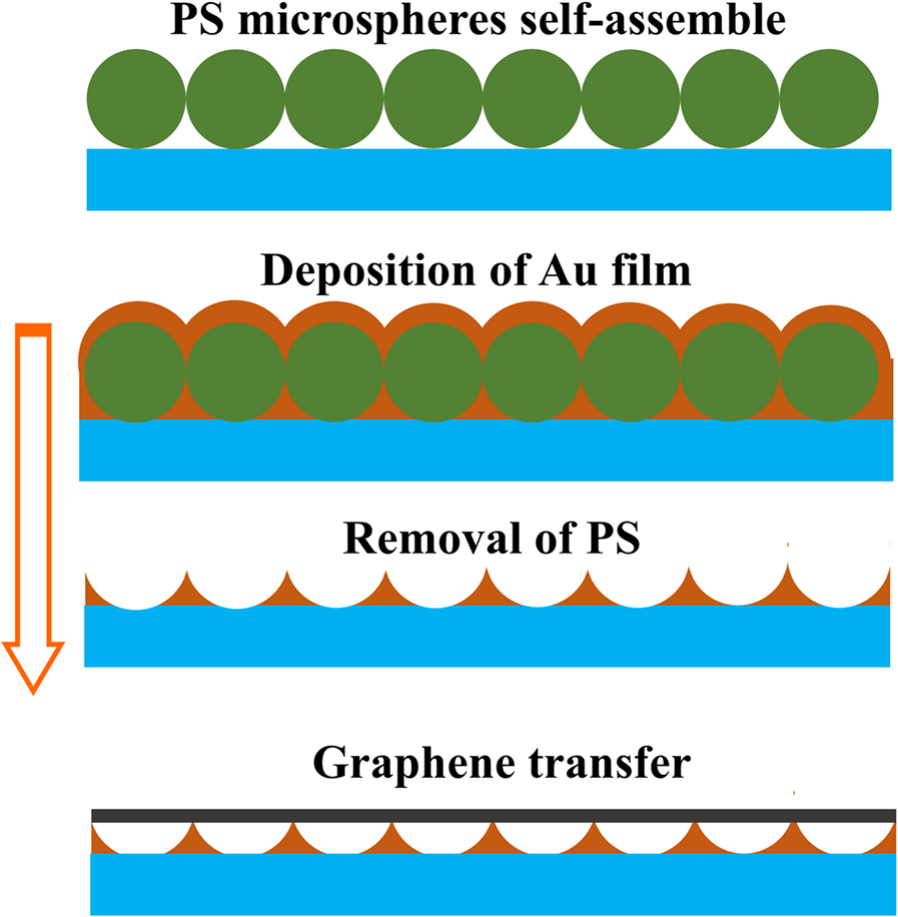
Schematic illustration of fabrication processes for the ordered Au nanostructures with graphene coverage

Colloidal microspheres of polystyrene (PS) (2.5 wt. %) with a diameter of 500 nm were purchased from Alfa Aesar and self-assembled to form a hexagonal close-packed (hcp) monolayer on the SiO_2_/Si or quartz substrates. Prior to the hcp alignment of the PS microspheres, the target substrates were firstly immersed in piranha solution (98 % H_2_SO_4_:37 % H_2_O_2_ = 7:3) for 3 h to achieve a completely hydrophilic surface, which contributed to the adhesion of PS microspheres to the substrates surface. Two parallel hydrophilic Si wafers with a distance of ~100 μm were mounted on the dip coater, and two or three drops of the PS sphere suspension were dropped into the gap of the Si wafers. With one Si substrate fixed, the other parallel Si was lifted with a constant speed of approximately 500 μm/s. The monolayer PS microspheres were ultimately formed on the hydrophilic surface of Si. Then, the PS monolayer was used as a template for the deposition of the Au film. The Au films were deposited on the PS template using the ion beam-assisted deposition (IBAD) system with a Kaufmann ion source. The size and shape of Au nanostructures were adjusted by varying the nominal thicknesses of the initial Au films ranging from 15 to 40 nm. After ultrasonic washing in acetone for 30 min, the PS microspheres together with the upper Au on them were completely removed and the ordered and size-tunable bottom Au nanostructures were formed. The few-layer graphene (FLG) films were synthesized on the Cu foils by low-pressure CVD in a tubular quartz reactor, using methane as the carbon source under H_2_ and Ar atmosphere at 1000 °C. Then, they were transferred to cover the Au nanostructures after the Cu foils were removed by wet chemical etching. The schematic illustration of fabrication processes for the ordered Au nanostructures with graphene coverage is shown in Fig. 1.

### Characterization

Surface morphologies of the ordered Au nanostructures with various shapes and sizes were characterized by atomic force microscopy (AFM, NT-MDT solver P47). The Au nanostructures with graphene coverage were investigated by field-emission scanning electron microscopy (SEM, Hitachi FE-S4800). Raman spectra of graphene were recorded with a Horiba LabRAM HR800 spectrometer using the 514-nm excitation line from an Ar ion laser. For both SEM and Raman measurements, the SiO_2_/Si substrates were adopted. The ultraviolet (UV)-visible absorption spectra of the ordered Au nanostructures were measured as a function of the incident wavelength using a Varian Cary 5000 spectrophotometer in a double-beam mode. The quartz substrate was adopted for the UV-vis measurement of the Au nanostructures with and without graphene, respectively.

## Results and Discussion

**Fig. 2:**
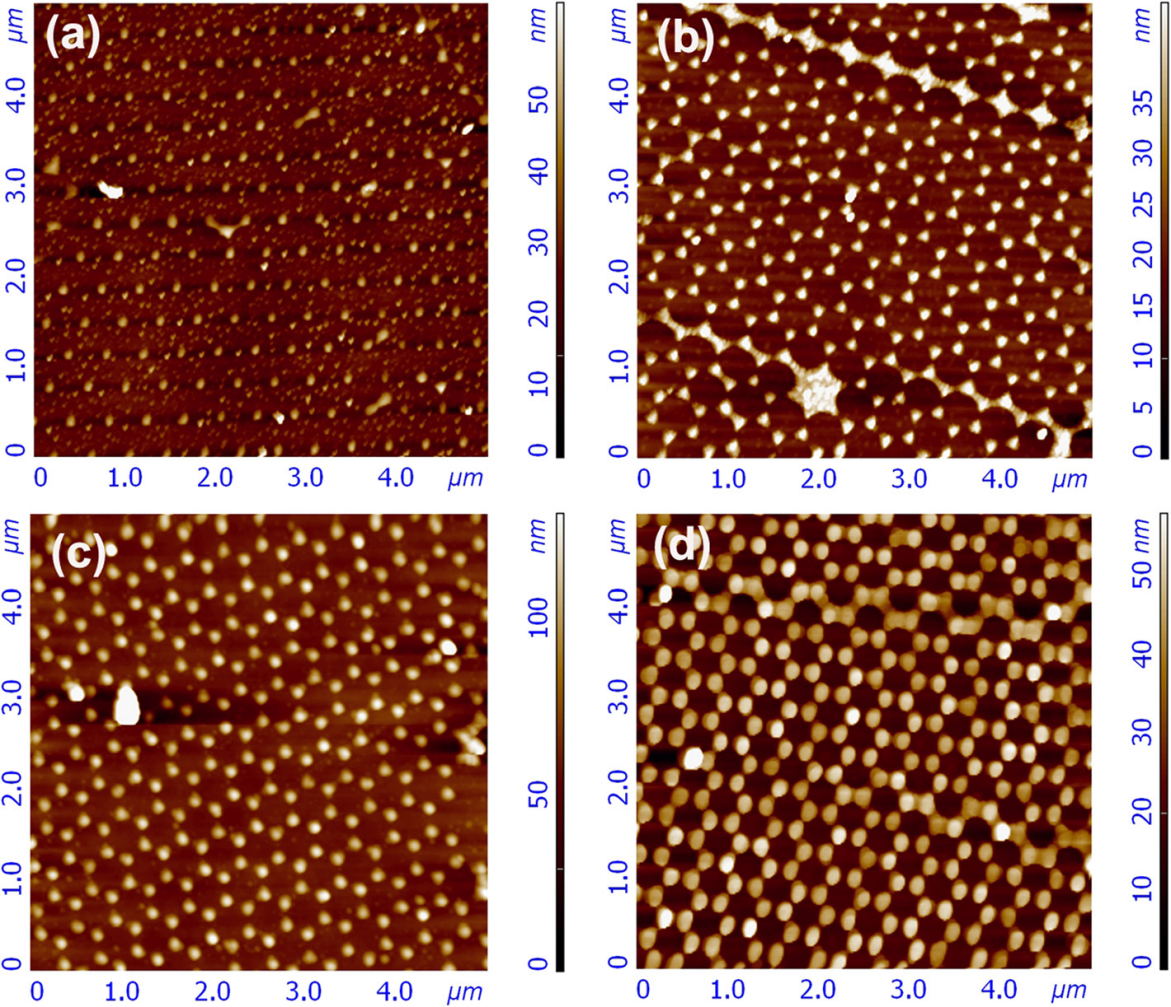
AFM images of the Au nanostructures with the initial thicknesses of **a** 15 nm, **b** 20 nm, **c** 30 nm, and **d** 40 nm

**Fig. 3:**
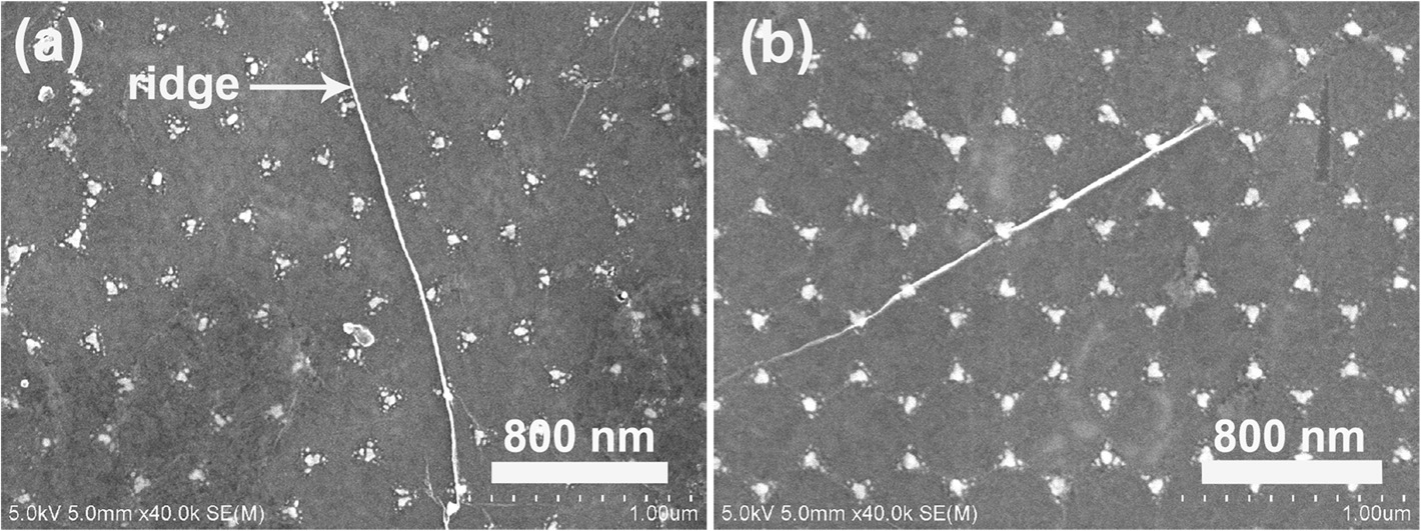
Typical SEM images of the Au nanostructures with initial thicknesses of **a** 20 nm **b** 30 nm covered by the graphene film

Figure 2 shows the AFM images of Au nanostructures with different sizes and shapes after removing the PS microspheres. Obviously, the long-range hexagonal order inserted from the original PS template is conserved. When the initial thickness of the Au film is 15 nm, the shape of the Au nanostructures is sphere-like nanoparticles, with an average width of ~100 ± 4.5 nm. Another point we have to notice from the SEM images is that the shape of the Au nanostructures gradually changed from more sphere-like nanodots to sharp triangles with the increase of the initial thickness of the Au film from 15 to 20 nm. The effect of the shape changes was also quantified by evaluating the circularities of the individual nanostructures, defined as the ratio of the square of the perimeter to 4*πA*, where *A* is the area of the particular nanostructure. The circularity should be 1.654 for a regular triangle and should approach 1 for a perfect circle [13]. The resultant values of the Au nanostructures with and without graphene coverage are listed in Table 1. From the table, we can distinctly see that with the deposition time increasing from 10 to 30 min (corresponding initial thickness from 15 to 40 nm), the circularity of the Au nanostructures first increases and then decreases, and the average width changes from 100 ± 4.5 to 140 ± 7.8 nm. As the diameter of the PS sphere is 500 nm, the gap (inscribed circle) among the PS spheres is approximately 77 nm. When the initial thickness of the Au film is only 15 nm, the Au film cannot cover the whole gap surface. Due to the different thermal expansion coefficients between the Au films and the substrate, when the initial thickness of the Au film is ~15 nm, the compressive stress induced by the Ostwald ripening mechanism would cause the Ag films to form isolated nanoparticles. With the increase of the initial thickness of the Au film to 20 nm, the whole gap among the PS nanospheres can be approximately filled, leading to shape transformation of the Au nanostructures from nanodots to triangles. When the initial thickness of Au was further increased to 40 nm, the whole gap of the PS spheres can be fully filled and the shape of the Au nanostructures changes to nanospheres due to the larger thickness of Au. The typical SEM images of 15- and 20-nm Au nanostructures covered with graphene are presented in Fig. 3. It is clear that the continuous graphene film has been successfully transferred on the surface of Au nanostructures, and the electron beam can easily penetrate through the atomically thin graphene to display the underlying Au nanostructures. The ridges and cracks formed on the graphene surface during the wet transfer processes can be also distinctly observed.

**Table 1:** Circularity, width, and resonance position of Au LSP with and without graphene coverage as a function of the initial thicknesses of the Au film

Initial thickness of Au film (nm)	Circularity	Width (nm)	LSPR without graphene (nm)	LSPR with graphene (nm)
15	1.09 ± 0.02	100 ± 4.5	530	554
20	1.21 ± 0.01	120 ± 5.4	542	562
30	1.13 ± 0.02	128 ± 6.7	547	564
40	1.06 ± 0.04	140 ± 7.8	553	570

**Fig. 4:**
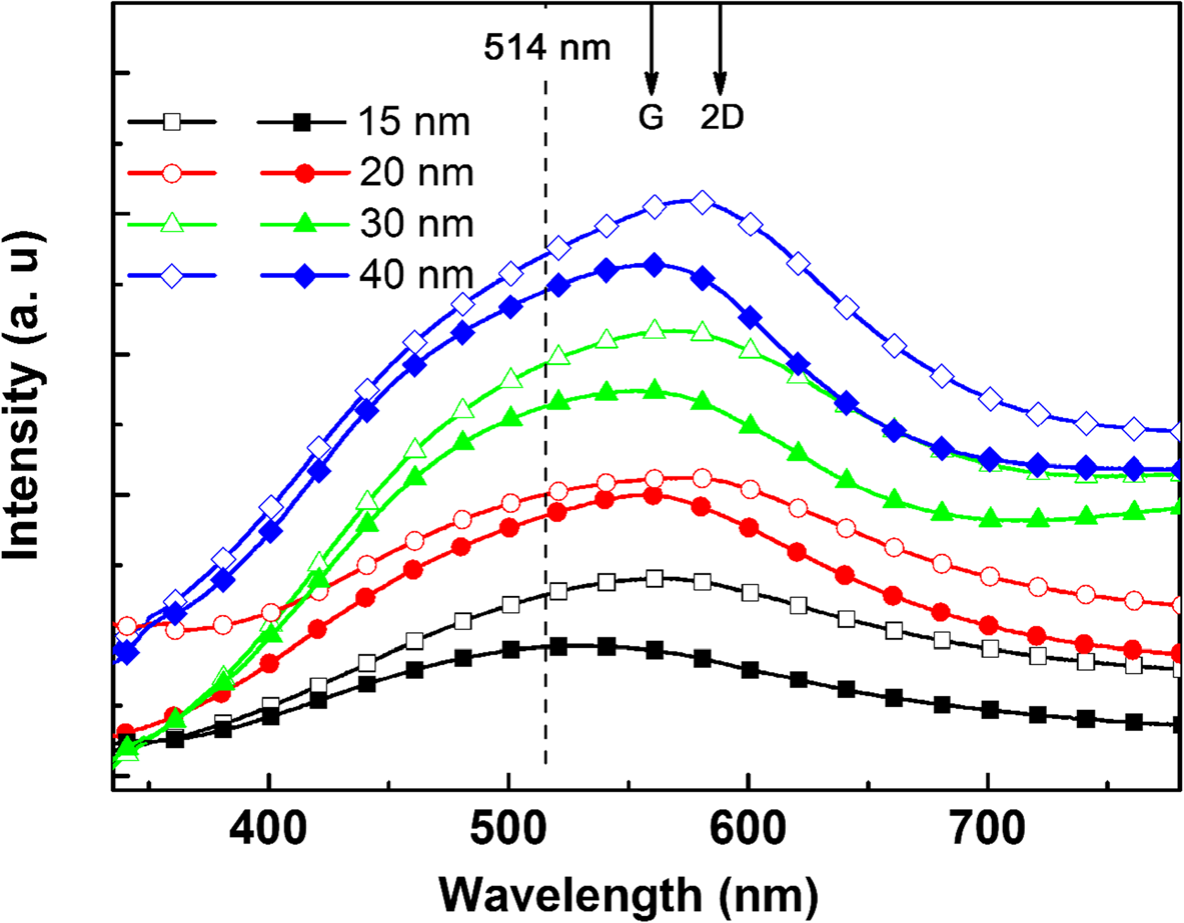
UV-vis absorption spectra of the various-sized Au nanostructures with (*open symbols*) and without (*solid symbols*) graphene coating. The Raman excitation wavelength of 514 nm is shown as a *vertical dashed line*, together with the corresponding wavelength of the G and 2D modes of graphene

To investigate effects of graphene on the plasmonic Au nanostructures, LSP properties of Ag nanostructures with and without graphene coating were characterized by a UV-visible absorption spectrophotometer. As shown in Fig. 4, the absorption spectra of the Au nanostructures on quartz substrates exhibit a wide plasmonic resonance peak varying from sample to sample, indicating that the LSP resonance of Au can be tuned by adjusting the size and shape of the Au nanostructures. The absorption intensity after graphene coverage increases slightly which can be ascribed to the absorption of the graphene film. Meanwhile, an obvious redshift and broadening of the Au LSP peak can also be clearly observed for all samples with graphene coating. Resonance positions of Au LSP before and after graphene coverage are also summarized in Table 1, and we can clearly see that the Au LSP resonances exhibit an ~20-nm redshift. On the other hand, the full width at half maximum (FWHM) of Au nanostructures with graphene also shows a distinct broadening compared with their counterparts without graphene. As the sizes of the nanostructures are smaller than the wavelength of incident light, the quasistatic model can be used to describe the position of the resonance peak. When irradiated by light, the conduction electrons will move and resonate with a specific frequency which is referred to as plasmon frequency of the particle dipole. According to the quasistatic analysis, the absorption peak corresponds to the dipole surface plasmon resonance for the ordered Au nanostructures on the quartz substrates [14]. When the particle size increases, the conduction electrons cannot all move in phase anymore, that is to say, the quadrupole resonances may occur except for the dipoles. The optical spectra of Au nanoprisms display in-plane dipole and quadrupole resonances. And the interaction of the dipole and quardrupole leads to a reduction of the depolarization field, which is the origin of the redshift of Au LSP resonances [14].

The above results also indicate that the Au LSPs are also strongly affected by the presence of graphene, which can be attributed to the coupling between the graphene film and the localized electromagnetic field of the Au nanostructures. As the incident light is perpendicular to the surface of the Au nanostructures, the incident electric field is parallel to the sample surface and has no vertical component, and only the lateral electron oscillations within the Au nanostructures can be induced. When the LSPs of Au nanostructures with graphene coating are excited, the image dipoles or quadrupoles within the graphene sheet which are antiparallel to the dipoles or quadrupoles in Au will be formed [5, 12]. The presence of the antiparallel image dipoles and quadrupoles can reduce the internal electric field in the Au nanostructures, which results in the redshift and broadening of LSP resonance peaks for the Au nanostructures with graphene.

Theoretical calculation based on the dipole approximation (results not shown here) has been conducted to understand the redshift of Au LSPs after graphene coverage. In the calculation, the Au nanosphere is utilized as a representative of the Au nanostructures and is placed above a transparent substrate. The thickness of the substrate is assumed to be semi-infinite, and absorption of the substrate is completely omitted. The dielectric constant of graphene is based on an assumption that the optical response of every graphene layer is given by optical sheet conductivity [5, 9], and the dielectric constant of Au can be found in the literature [15]. The graphene sheet covers the surface of the Au nanosphere, which is treated as a single dipole. Considering presence of the antiparallel dipole in graphene, the polarizability *α* of the Au nanosphere can be written as [9, 16]

1 $$ \alpha =4\pi {a}^3\left(\frac{\varepsilon_1-{\varepsilon}_2}{\varepsilon_1+2{\varepsilon}_2}\right){\left[1-\beta {\left(\frac{a}{2d}\right)}^3\left(\frac{\varepsilon_1-{\varepsilon}_2}{\varepsilon_1+2{\varepsilon}_2}\right)\left(\frac{\varepsilon_3-{\varepsilon}_2}{\varepsilon_3+{\varepsilon}_2}\right)\right]}^{-1} $$

where *α* is the polarizability of Au nanosphere, *β* is 1 for the lateral electric field, and *d* is the distance between the center of the Au nanosphere and the graphene sheet. *ε*_1_, *ε*_2_, and *ε*_3_ are the dielectric constants of Au, ambient, and graphene, respectively [16]. The absorption efficiency of Au *Q*_abs_ can be expressed as *Q*_abs_ = [*k*/*πa*^2^]Im(*α*). The calculated results of *Q*_abs_ for a single 30-nm Au nanosphere with and without graphene coating show that the absorption peak of the Au nanosphere exhibits a slight redshift (about 20 nm) after graphene coating, which is in good agreement with the experimental data. The discrepancy of the absorption results between calculation and experimental results can be ascribed to the simplified assumptions during the calculation such as the semi-infinite substrate, the dipole approximation, the homogeneous particle size, and the crystal perfection of the Au nanosphere [5, 10].

**Fig. 5:**
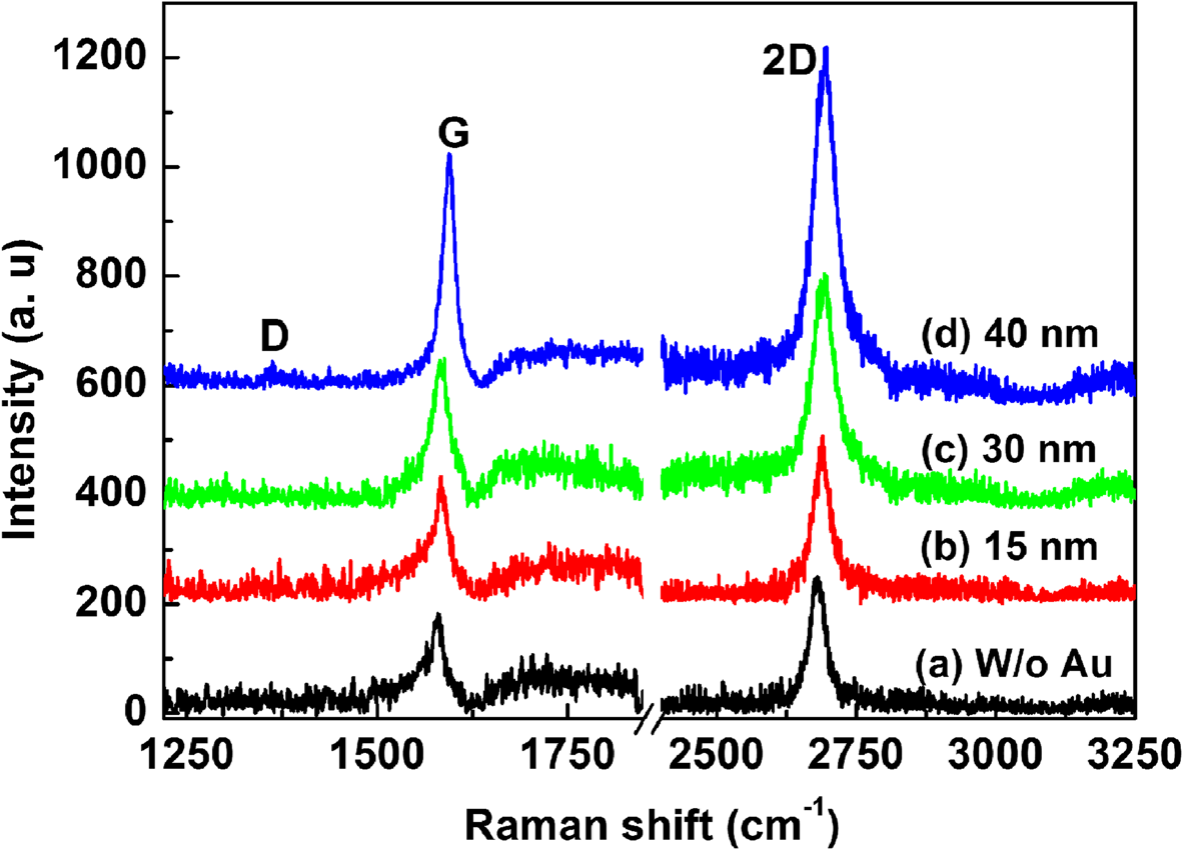
Raman spectra of the graphene films (*a*) without Au nanostructures and on the surface of the ordered Au nanostructures with initial thicknesses of (*b*) 15 nm, (*c*) 30 nm, and (*d*) 40 nm

To shed light on the effects of plasmonic ordered Au nanostructures on the Raman properties of graphene, the Stokes Raman spectra of pristine graphene and graphene transferred on differently shaped and sized Au nanostructures were measured and the corresponding results are shown in Fig. 5. The Raman spectrum of the pristine graphene reveals the well-known D peak (1353 cm^−1^), G peak (1590 cm^−1^), and 2D peak (2690 cm^−1^). The G peak originates from the first-order Raman scattering process, while the 2D peak is due to a double-resonance intervalley Raman scattering process [17, 18]. The nearly negligible intensity of the D peak indicates few structural and crystalline defects in the CVD graphene. The Raman peak of graphene on top of the 100-nm Au nanostructures exhibits almost the same amplitude with its counterpart on the SiO_2_/Si substrate. It is apparently revealed that the intensities of graphene G and 2D peaks are significantly enhanced with increasing size of the ordered Au nanostructures from 100 to 140 nm. With increasing size of the ordered Au nanostructures to 140 nm, the Raman peak intensities of graphene on top of Au display an enhancement factor of ~3-fold for the G peak and 2.5 for the 2D peak. Therefore, the SERS of graphene caused by the ordered Au nanostructures was distinctly observed, and the underlying mechanism will be discussed in detail later.

Generally, both the LSP and charge transfer can play a role in the SERS of graphene modes, since the SERS electromagnetic enhancement results from the Raman excitation and coupling with the LSP of nanostructures, while the metal-induced charge transfer can lead to the chemical resonance enhancement [19]. In our case, the enhanced Raman intensity of graphene/Au nanostructures is mainly attributed to the electromagnetic field enhancement induced by the plasmonic resonance of Au LSP. As illustrated in Fig. 5, the enhancement factor of both G and 2D Raman peaks increase with the size of the Au nanostructures, which is an indication of the electromagnetic mechanism. The rather qualitative image presented in Fig. 5 coincides well with a theoretical model that has been proposed for Raman enhancement in the graphene-nanoparticle hybrid [10, 20]. According to this model, the Raman enhancement due to the stand-alone nanoparticle is given by

2 $$ \frac{\varDelta {I}_{\mathrm{SERS}}}{I_0}\approx \frac{3}{28}\sigma {Q}^2\left(\omega \right){Q}^2\left({\omega}_s\right){\left(\frac{a}{h}\right)}^{10} $$

where Δ*I*_SERS_ is the increase in Raman intensity with respect to its original intensity *I*_0_, *σ* is the relative cross-sectional area of the nanoparticle, *Q*(*ω*) is the plasmonic enhancement from the Mie theory with *ω*_s_ representing the Stokes Raman frequency, *α* is the particle radius, and *h* represents the separation between the particle center and the surface of the graphene sheet. From Eq. 2, we can easily deduce that the Raman enhancement scales with the cross section of the metallic nanostructures, with the fourth power of the Mie enhancement, and inversely with the tenth power of the distance between the graphene and nanoparticle center. Therefore, the Raman scattering enhancement with increasing particle size (as shown in Fig. 5) can be mainly ascribed to the plasmonic absorption profile *Q*(*ω*) of the nanoparticle. The shapes of the particles vary with different initial thicknesses of the Au film, although majority of Au nanostructures can be treated as spheres or triangle prisms. Experiments of the absorption spectra have shown that the plasmonic resonance positions for the ordered Au nanostructures with various size ranges from 100 to 140 nm lie around the excitation laser wavelength (514 nm, dotted line in Fig. 4); therefore, the incident laser beam excites the Au LSPs which will form a strong localized electromagnetic field around the nanostructures. As the graphene sheet is in close vicinity of the Au nanostructures, the electric field will penetrate into the graphene sheet, and an enhanced electromagnetic field will be formed on the graphene surface, although the antiparallel image dipole will form and reduce the local electric field around the Au nanostructures [12]. Ultimately, the *Q*(*ω*) in Eq. 2 will be significantly improved, and the SERS signal is greatly enhanced. Moreover, the enhancement factor of the G and 2D peaks gradually increases with the size of the Au nanostructures, as shown in Fig. 5, which can be interpreted as follows: The corresponding wavelengths of the G and 2D peaks of graphene are consistent with those of the Au LSP resonances (see Fig. 4). Therefore, the enhanced local electromagnetic field induced by Au LSPs contributes to the improved Raman signal. On the other hand, with increasing average radius of the ordered Au nanostructures, *ΔI*_SERS_ will be distinctly increased which can be simply estimated from Eq. 2.

Now we turn towards the nature of the chemical interaction between the ordered Au nanostructures and graphene, which is another physical mechanism for the SERS especially for the graphene-nanoparticle hybrid system [3, 19]. From the Raman spectrum, we find that the G peak position spectrally shifts from 1350 to 1360 cm^−1^ for graphene that was transferred on the surface of the ordered Au nanostructures while the 2D peak position almost remains constant. It has been reported that the G peak of graphene is blueshifted for both electron and hole doping, while the 2D peak is redshifted for electron doping and blueshifted for hole doping [18, 21]. In this case, graphene is in direct contact with the ordered Au nanostructures, and the work function of Au (5.0 eV) is nearly the same with that of graphene (4.8 eV). Considering that there are huge amounts of electrons for the Au nanostructures, electron transfer from Au to graphene will occur, leading to an electron doping for graphene; thus, the G peak is blueshifted and the 2D peak is slightly redshifted. On the other hand, we deduce that graphene is under compressive strain when it is directly transferred on top of Au as some ridges appear on the surface of graphene (clearly in Fig. 3); hence, both the G and 2D peaks exhibit a blueshift trend [21]. As a result, both strain and doping effects lead to a slight blueshift for the G peak and negligible shift for the 2D peak position. However, we consider that charge transfer is not the dominant mechanism for the enhanced Raman intensity since the Raman intensity is increased with the size of Au nanostructures and the charge transfer effect should be independent on the Au size.

## Conclusions

In summary, the coupling between graphene and LSPs of ordered and size-controllable Au nanostructures has been investigated systematically by directly transferring graphene on the surface of the Au nanostructures. The absorption spectra of Au exhibit a redshift of ~20 nm after graphene coverage, which can be ascribed to the plasmonic coupling between the Au LSPs and graphene. On the other hand, the graphene SERS is significantly observed, and intensities of the G and 2D peaks increase with increasing size of the ordered Au nanostructures. The electromagnetic plasmonic effect rather than the charge transfer mechanism is considered to be the dominant mechanism for the SERS effect of graphene. We believe the results are beneficial not only for further understanding the coupling mechanism between graphene and the ordered metallic nanostructures, but also for developing plasmonic graphene-based optoelectronic devices.
